# Amplitude Manipulation Evokes Upper Limb Freezing during Handwriting in Patients with Parkinson’s Disease with Freezing of Gait

**DOI:** 10.1371/journal.pone.0142874

**Published:** 2015-11-18

**Authors:** Elke Heremans, Evelien Nackaerts, Griet Vervoort, Sarah Vercruysse, Sanne Broeder, Carolien Strouwen, Stephan P. Swinnen, Alice Nieuwboer

**Affiliations:** 1 Neuromotor Rehabilitation Research Group, Department of Rehabilitation Sciences, KU Leuven, Leuven, Belgium; 2 Center for Statistics, Hasselt University, Hasselt, Belgium; 3 Movement Control and Neuroplasticity Research Group, Department of Kinesiology, KU Leuven, Leuven, Belgium; University of Tuebingen, GERMANY

## Abstract

**Background:**

Recent studies show that besides freezing of gait (FOG), many people with Parkinson’s disease (PD) also suffer from freezing in the upper limbs (FOUL). Up to now, it is unclear which task constraints provoke and explain upper limb freezing.

**Objective:**

To investigate whether upper limb freezing and other kinematic abnormalities during writing are provoked by (i) gradual changes in amplitude or by (ii) sustained amplitude generation in patients with and without freezing of gait.

**Methods:**

Thirty-four patients with PD, including 17 with and 17 without FOG, performed a writing task on a touch-sensitive writing tablet requiring writing at constant small and large size as well as writing at gradually increasing and decreasing size. Patients of both groups were matched for disease severity, tested while ‘on’ medication and compared to healthy age-matched controls.

**Results:**

Fifty upper limb freezing episodes were detected in 10 patients, including 8 with and 2 without FOG. The majority of the episodes occurred when participants had to write at small or gradually decreasing size. The occurrence of FOUL and the number of FOUL episodes per patient significantly correlated with the occurrence and severity of FOG. Patients with FOUL also showed a significantly smaller amplitude in the writing parts outside the freezing episodes.

**Conclusions:**

Corroborating findings of gait research, the current study supports a core problem in amplitude control underlying FOUL, both in maintaining as well as in flexibly adapting the cycle size.

## Introduction

Freezing in Parkinson’s disease (PD) is a sudden, variable, and often unpredictable transient break in movement [1]. This phenomenon has been studied widely during gait, as freezing of gait (FOG) is known to be one of the most disabling symptoms in patients with advanced PD. Freezing episodes were also reported to occur during speech and upper limb movements with striking similarities in the disrupted movement output [2]. Although these problems can severely impact on patients’ work, hobbies and self-care, far less research has been performed on such types of non-gait freezing.

Freezing has been difficult to study as it is challenging to elicit in a laboratory setting [3]. This is most likely due to the changes in gait pattern when patients are aware that they are being evaluated resulting in heightened arousal and a potential Hawthorne effect [4–6]. Recently, several tests were shown to provoke gait freezing in a lab setting, including rapid 360° turns, gait in combination with dual tasking or small-amplitude steps [7–9]. In contrast, sensitive methods to assess freezing episodes during hand movements are sparse.

Freezing of the upper limbs (FOUL) in PD was investigated previously during a 15 second finger tapping task at maximum speed and during point-to-point hand movements with a magnetic mouse [10, 11]. These previous systems classified freezing in the upper limbs based on a lack of change in movement amplitude of at least one second [10, 11]. Recent studies in gait, however, indicate that freezing is not always characterized by a complete cessation of movement, but rather by a lack of efficient movement cycles preceded by a reduction in movement amplitude and/or an irregular movement frequency [12–14]. In line with this definition, upper limb freezing episodes were identified in persons with PD during bimanual tasks that were performed in the off-phase of the medication cycle [12, 13]. So far, however, it is unclear which task constraints provoke and explain FOUL. Whereas Vercruysse et al. [13] found that upper limb motor blocks mainly occurred during finger tapping at small amplitude, other studies reported no differences in FOUL frequency between small- and large-amplitude conditions [12, 15]. During gait, freezing has been shown not only to be provoked by walking at small steps [7, 8] but even more by complex gait trajectories, requiring patients to adapt gait to changes in the environment such as turns, obstacles or doorway passages [3, 16]. This may be due to the specific difficulties that PD patients with gait freezing experience during set switching, which has been defined as the ability to flexibly alter one’s behavior when relevant changes occur in the predefined goal or in the environment [17]. These findings in gait research led to the hypothesis that, in a similar way, FOUL during writing may be triggered by imposing movement amplitude adaptations to changing task requirements rather than by solely asking patients to write at small versus large size. Therefore, in the current study, PD patients with and without FOG, who were matched for disease severity were investigated while making up- and downstroke writing-like movements at variable sizes, requiring gradual changes in movement size to make the transition from small to large size writing and vice versa. This innovative paradigm allowed us to test two contrasting hypotheses: 1) if freezing is caused mainly by a decreased ability in set switching, the majority of FOUL episodes would occur in the parts where gradual changes in size have to be made; 2) if freezing, on the other hand, results from difficulties to generate and maintain effective movement amplitudes, most FOUL episodes are expected to occur in the continuous trajectories in between the parts requiring amplitude changes. To increase the ecological value of the task, a unimanual task was chosen which is close to actual writing and the task was performed in the on-phase of the medication.

## Methods

### Participants

Thirty-four patients with clinically defined PD were recruited from the Movement Disorders clinic of the University Hospitals Leuven and were grouped according to item 3 of the new freezing of gait questionnaire (NFOG-Q) [18] into freezers (PD+FOG; n = 17) and non-freezers (PD-NoFOG; n = 17). PD+FOG and PD-NoFOG were matched a priori by group according to age and disease severity as measured by the Hoehn and Yahr (H&Y) scale and Movement Disorders Society-sponsored Unified Parkinson’s Disease Rating Scale (MDS-UPDRS) part III [19, 20]. Ten age-matched persons without any neurological or musculoskeletal dysfunction participated as controls. All participants were right-handed, as measured by means of the Edinburgh Handedness Inventory [21]. Demographics and clinical characteristics of all participants are specified in Table 1. Inclusion criteria for the patients with PD were (i) idiopathic PD, diagnosed according to the United Kingdom PD Society Brain Bank criteria [22] and (ii) H&Y stage I to III. Exclusion criteria were i) the presence of a deep brain stimulator, ii) the presence of dementia (score < 24 on Mini Mental State Examination (MMSE)) [23], iii) comorbidity affecting upper limb function, iv) the presence of depression or other neurological disorders and v) problems in vision hindering writing. The study design and protocol were approved by the local Ethics Committee of the KU Leuven and were in accordance with The Code of Ethics of the World Medical Association (Declaration of Helsinki, 1967). After complete explanation of the study protocol, written informed consent was obtained from all participants prior to participation in the experiment.

**Table 1:** Clinical characteristics of patients with Parkinson with and without FOG.

Parameter	PD+FOG	PD-FOG	Controls	*P*-value
Age (years)	64.5 (8.6)	64.6 (9.1)	65.8 (8.4)	0.85
Edinburgh Handedness Inventory	100 (80–100)	100 (90–100)	100 (90–100)	0.89
MMSE (0–30)	28 (28–29)	28 (26–30)	29 (29–30)	0.15
Disease duration (years)	10.3 (5.5)	5.8 (4.5)	NA	0.01*
MDS-UPDRS-III (0–108)	35.9 (14.8)	32.4 (11.7)	NA	0.44
H&Y (0-V)	2 (2–2)	2 (2–2)	NA	0.34
NFOG-Q (0–23)	15 (8–19)	0 (0–0)	NA	<0.01*
LED (mg/24h)	398 (256)	399 (259)	NA	0.21

Abbreviations: MMSE = Mini Mental State Examination; MDS-UPDRS-III = Movement Disorders Unified Parkinson’s disease rating scale part 3; H&Y = Hoehn and Yahr stage; NFOG-Q = New freezing of gait questionnaire; LED = levodopa equivalent dose.

Results are presented as mean and 1 standard deviation for normally distributed data and as the median and interquartile range (Q1-Q3) for non-normally distributed data. NA = not applicable.

* indicates p-value <0.05.

### Experimental procedure and tasks

Before performing the experimental task, all participants were assessed by means of the MMSE and Edinburgh Handedness Inventory. In addition, motor function of the patients with PD was evaluated by means of the MDS-UPDRS part III and NFOG-Q. Both the clinical examination and writing tests were examined while patients were in the on-phase of the medication cycle approximately 1–1.5 hours after medication intake.

**Fig 1:**
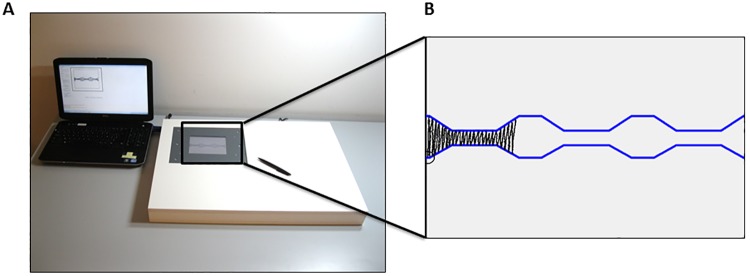
Experimental setup (A) and writing task (B). The writing task included continuous writing at large (2 cm) and small (0.6 cm) amplitudes as well as gradual increases (from 0.6 cm to 2.0 cm) and decreases (from 2.0 to 0.6 cm) in writing size.

Writing data were recorded on a touch-sensitive writing tablet with a sampling frequency of 200 Hz and spatial resolution of 32.5 μm [24, 25] (Fig 1A). This tablet was built into a larger wooden frame, supporting the arm and hand. Participants were provided with real time feedback of their writing-like movements by means of a flat screen display. All tests were performed in a quiet room while sitting at a table on a height-adjustable chair. The tests included five trials consisting of execution of alternating upstroke and downstroke writing-like movements with the right hand at varying amplitudes. Each trial included continuous writing at large (2 cm) and small (0.6 cm) amplitudes as well as gradual increases (from 0.6 cm to 2.0 cm) and decreases (from 2.0 to 0.6 cm) in writing size (Fig 1B). The requested size was indicated by a blue target zone with a width of 2 mm. Before the actual test started, a demonstration was given by the experimenter and participants were provided with a practice trial. Next, they were asked to perform the task at comfortable speed until the end of the 13 cm writing trajectory was reached or, if they did not reach the end of the trajectory in time, until the end of the 1-minute time span that was provided per trial. All participants performed five trials with 6 second breaks in between. Each trial was preceded by a figure instructing the participant on the upcoming task and initiated with a starting tone. No other auditory cues were given.

### Data processing

**Fig 2:**
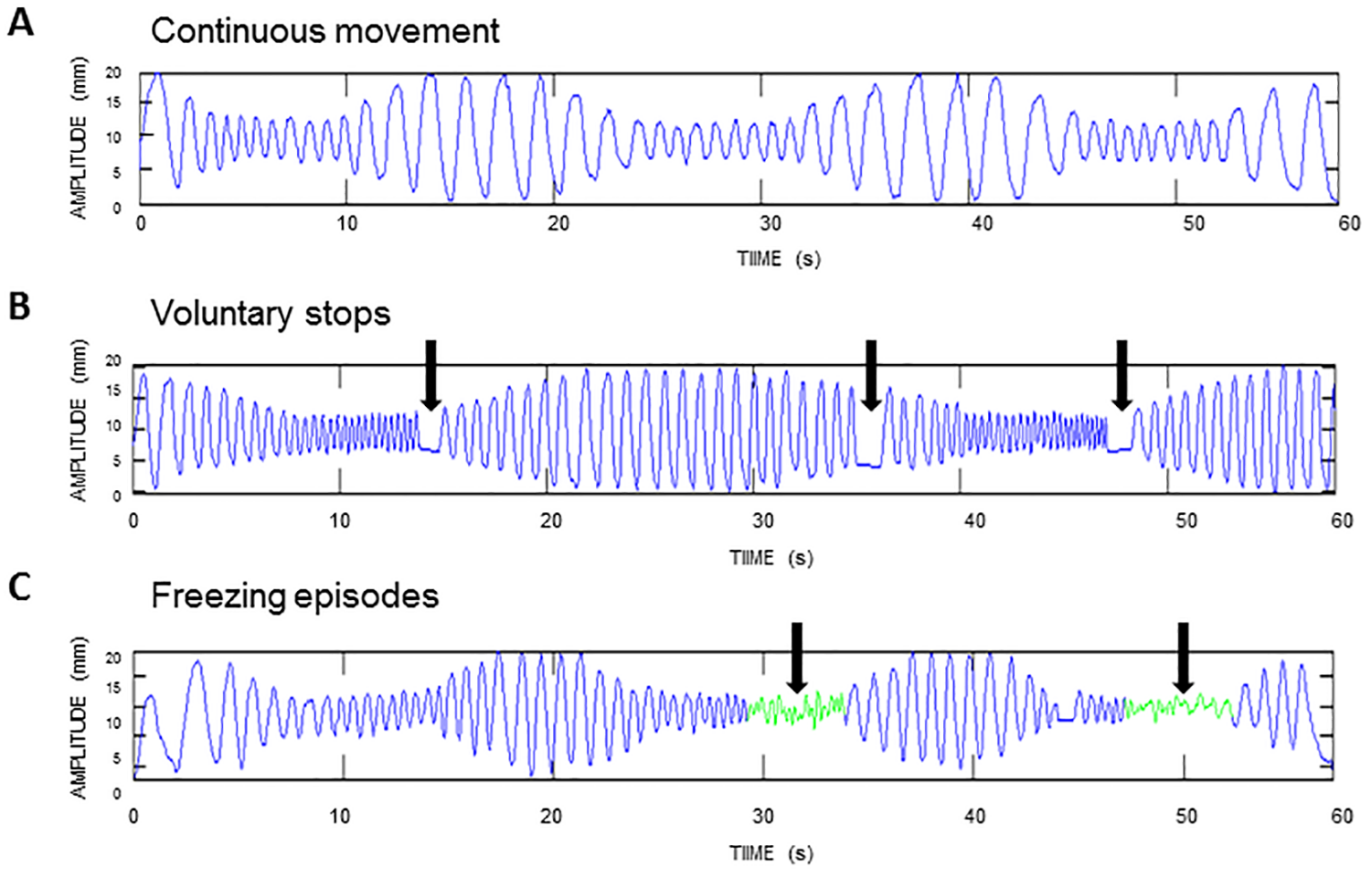
Representative example of a writing trace without voluntary stops or freezing episodes (A), a writing trace with voluntary stops (B) and a writing trace with two freezing episodes (depicted in green) (C).

All trials were analyzed for the presence of FOUL by two independent raters, and in case of disagreement between those raters, by an additional third rater. In line with gait, handwriting freezing was defined as an involuntary stop or clear absence of effective writing movements during at least 1 second. Freezing episodes were visually determined as ineffective movement cycles preceded by or characterized by a decreased writing amplitude (<50% of the target amplitude), irregular cycling frequency and/or increased freezing index, after processing in Matlab R2011b (Mathworks) [13]. Movement amplitude and cycle frequency were determined for each movement cycle using the difference between the maximum and minimum amplitude and by taking the inverse of the time that elapsed between successive peak positions (frequency). The freezing index was defined as the power in the freeze band (3–8 Hz) divided by the power in the normal motion band (0.5–3 Hz) [26]. Voluntary stops were documented during data collection to ensure that FOUL episodes were not falsely identified as intentional movement arrests and vice versa. As well, voluntary stops were distinguished from FOUL episodes as they were not preceded by a reduction in amplitude and irregularity in cycle frequency. Fig 2 illustrates examples of a normal trace, a trace with voluntary stops and a trace with FOUL. For each participant, the number of freezing episodes, duration of each episode and the part in the trajectory (i.e., small, large, decreasing size, increasing size) where the freezing episode started was determined. A binary FOUL score was calculated, with a score of 1 indicating the occurrence of at least one freezing episode, and a score of 0 the absence of FOUL. Similarly, a binary FOG score was determined, with a score of 1 indicating a score of 1 or higher on the NFOG-Q.

After removing the kinematic data of the parts where freezing occurred, the mean movement amplitude and speed were calculated per participant for each part of the writing trajectory separately. Movement amplitude was defined as the distance (in mm) between the local minima and maxima. For each up- and downstroke movement, the time to completion (s) was computed and used to calculate writing speed (cm/s). Trials where the mean amplitude or speed deviated more than 2 standard deviations from the group mean were considered artifacts and were removed from the analysis (<5% of the data).

### Statistical analyses

All analyses were performed using STATISTICA (Statistica Analysis Software, version 10) with significance levels of 0.05. Normality and equality of variance were checked for all variables. Clinical variables were compared between groups using a one-way analysis of variance (ANOVA) in case of normal distribution of the data and nonparametric Kruskal-Wallis or Mann-Whitney U tests in case of abnormality or a discrete nature of the outcome variable.

Spearman’s rank correlations were carried out to test the relationship between the occurrence of FOUL (yes/no score), the number of FOUL episodes, the occurrence of FOG (yes/no score), the score on the NFOG-Q and clinical outcomes within PD patients. The amplitude and speed of the kinematic trajectories outside the freezing episodes were compared between groups by means of a 3 x 4 repeated measures ANOVA with group (controls, PD-NoFOG, PD+FOG) as a between-subjects factor and size (small, large, decreasing size, increasing size) as a within-subjects factor. Tukey HSD post hoc tests were performed where appropriate. The parts with freezing were excluded from this analysis.

## Results

### Subjects

Patients with and without FOG were matched for age, gender, L-dopa equivalent dose (LED) intake, cognition and disease severity, as measured by the MMSE, UPDRS-III and H&Y stage. A significant difference between PD-NoFOG and PD+FOG was found for disease duration (p = 0.01) (Table 1). Significant differences in NFOG-Q scores (p<0.01) confirmed the validity of the subgroups. The control group consisting of 10 healthy participants was matched for age and cognition with both PD groups.

### Upper limb freezing episodes

**Fig 3:**
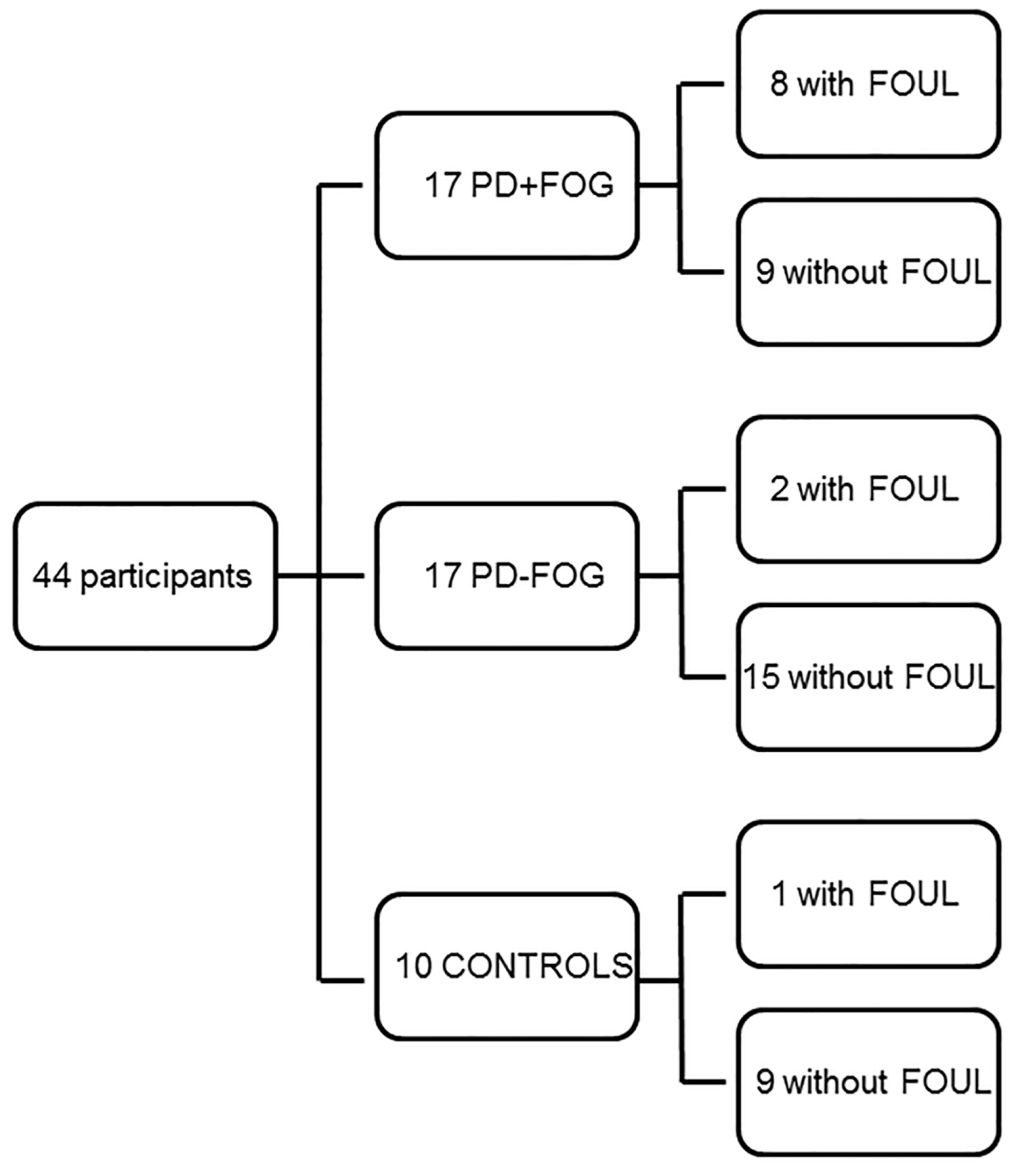
Schematic overview of the occurrence of FOG and FOUL in all participants.

In the total of 170 patient trials, 50 upper limb freezing episodes lasting longer than 1 second were detected in 32 trials. These episodes were detected in 10 patients, including 8 PD+FOG and 2 PD-NoFOG. In addition, 2 freezing trials with 1 freezing episode were detected in a control participant (Fig 3). Fifty-four (53.9) % of the FOUL episodes started in the small parts of the writing trajectory, 34.6% in the part where participants had to gradually decrease their writing size and only 7.7% and 3.8% in the large part and part where they had to increase their writing size, respectively. The duration of the FOUL episodes ranged from 1.0 to 9.8 s with a mean duration of 3.6 (±2.7) s. The freezing trials had a mean freezing index of 1.2 (± 0.6).

### Correlation between FOUL, FOG and clinical outcomes

All patients were divided into two groups: patients with and without FOUL. The binary FOUL score correlated significantly with the binary score of patients having FOG or not (r = 0.39; p<0.05) and with patients’ N-FOGQ score (ρ = 0.41; p<0.05). In line, there was a significant correlation between the number of upper limb freezing episodes per patient and the binary score of patients having FOG or not (ρ = 0.36; p<0.05) and with the NFOG-Q scores (ρ = 0.38; p<0.05). The patients’ binary FOUL and FOG scores, number of FOUL episodes and NFOG-Q scores did not correlate with disease severity, as measured by UPDRS-III and H&Y stages. Significant correlations, however, were found between disease duration and binary FOUL score (ρ = 0.49; p<0.05), number of FOUL episodes (ρ = 0.51, p<0.05), binary FOG score (ρ = 0.46, p<0.05) and NFOG-Q score (ρ = 0.45, p<0.05).

### Upper limb performance outside the freezing episodes

#### Writing amplitude

**Fig 4:**
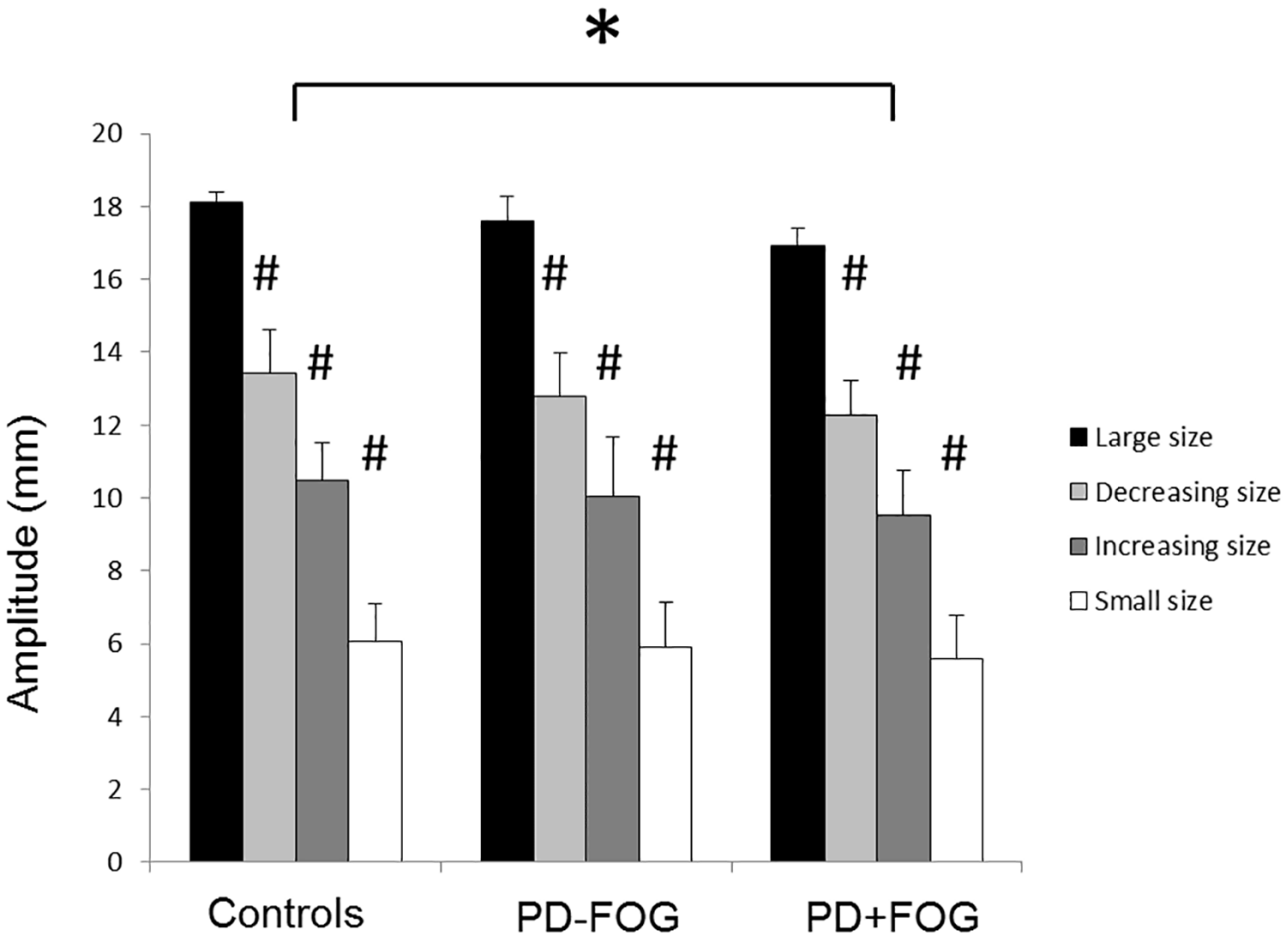
Writing amplitude (mm) per group and size. The main effect for group (indicated with *) shows a significant difference between PD+FOG and Controls. The main effect for Size (indicated with #) showed significant differences between all four sizes. Data are displayed as group means and standard deviations. Asterisks indicate significant differences at α<0.05.

A 3 x 4 ANOVA showed a significant main effect for group (F = 2,41) = 3.97; p = 0.03) and size (F(3,123) = 1612.8; p<0.01) (Fig 4). PD+FOG had significantly smaller amplitudes in the writing parts outside the freezing episodes (11.1 ± 4.3 mm) than controls (12.0 ± 4.5 mm). No significant differences were present between the PD-NoFOG (11.6 ± 4.4 mm) and both other groups. The main effect for size indicated that, as expected, all participants adapted their size to requested target sizes of the different parts of the figure (small parts: 5.8 ± 0.6 mm, large parts: 17.4 ± 1.2mm, parts with decreasing size: 12.7 ± 1.4 mm, parts with increasing size: 9.9 ± 1.1mm). Further analysis comparing patients with and without FOUL showed that patients with FOUL moved with significantly smaller amplitude in all parts than patients without FOUL (F(1,32) = 9.11; p<0.01).

#### Writing speed

Also for speed, a 3 x 4 ANOVA indicated significant main effects for group (F2,41) = 3.85; p = 0.03) and size (F(3,123) = 22.24; p<0.01). Post hoc analysis showed that the PD+FOG (2.5 ± 1.4 cm/s) wrote significantly faster than PD-NoFOG (1.49 ± 1.0 cm/s). The writing speed of the control group (2.00 ± 1.0 cm/s) did not differ significantly from both other groups. Post hoc analyses for size showed that all participants wrote faster when writing small compared to writing at large size and at decreasing size. Writing at increasing size was faster than writing at continuous large size and writing at decreasing size (p<0.01 for all comparisons). Subgroup analyses comparing PD patients with and without FOUL showed a tendency that patients with FOUL wrote faster than those without, but this effect did not reach significance (p = 0.10).

## Discussion

The aim of the current study was to test whether gradual changes in amplitude in contrast to sustained amplitude generation provoked upper limb freezing using a novel funnel-like writing paradigm. Even though patients were optimally medicated, upper limb freezing episodes were detected in ten out of 34 patients with PD of which 8 with FOG and 2 without FOG. The main result is that upper limb freezing episodes mainly occurred in the troughs of the funnel, where participants had to write continuously at small amplitude (0.6 cm) and in the preceding parts requiring gradual scaling down of the writing size. The occurrence of FOUL during writing at small size is in line with previous studies showing decreased coordination and increased probability of FOUL when making small and fast bimanual movements in PD patients with FOG in comparison to healthy controls [13, 15]. As well, it extends findings from gait research showing that an artificial reduction of step length in freezers provokes the same effects (i.e. increased number of freezing episodes, greater variability in step length) as observed in a variable environment when an automatic reduction and adaptation of step length is required [8]. This core amplitude problem also manifested as reduced movement amplitude outside freezing episodes. To date, it is unclear to what extent the freezing episodes and reduced amplitude in the non-freezing parts may share similar underlying pathophysiological mechanisms. In a recent review by Wu et al. [27], it was hypothesized that impaired motor automaticity may be an important reason underlying both freezing and bradykinesia.

A novel finding in the present study was that many FOUL episodes were also triggered in the parts of the writing trajectory where persons were forced to gradually decrease their writing size. The occurrence of FOUL in these parts may also offer evidence for the hypothesis that PD patients with FOUL experience difficulties in set switching, which involves alterations in behavior due to either motor or cognitive task constraints. A recent study showed that mainly PD+FOG experience a deficit in motor switching, which was defined as a change in stepping direction from one trial to the next [28]. However, in the current study, no increase in FOUL was found when patients had to gradually increase their writing size, contradicting the hypothesis that the freezing episodes are merely triggered by impaired motor switching. Alternatively, it was suggested that the key contributor to provoking FOUL is the shifting of attentional resources between both motor and sensory demands of a task [14]. It is likely that the amplitude decrease in the current writing task to move past the narrow passage required a shift from more automatic to more cognitively controlled writing, exceeding the limited resource capacity in the patients with FOUL. Similary, freezing-like events were provoked during gait when approaching a doorway, with greater frequency as the door width decreased [29]. The fact that this happened mostly in PD+FOG is in accordance to findings in gait showing that increased planning demands have a direct influence on movement control in PD+FOG only [30]. As well, studies using cognitive tasks showed specific deficits in executive functioning in PD patients with FOG [31, 32]. Freezers have more problems to discern whether, in a particular context, inhibition is needed or not, and have a failure to release such inhibitory responses once initiated [31]. Freezing-like behavior was triggered by navigating through a virtual environment (including narrowing doorways) using a hand-controlled button box if the task had to be performed under increased cognitive load [33]. In support of this finding, in a later study it was found that during a virtual reality gait task under high cognitive load, PD+FOG hyperactivated areas associated with cognitive control when contrasted with PD-NoFOG, such as the dorsolateral prefrontal cortex and parietal cortex [34].

The finding that 88.5% of the FOUL episodes occurred during writing at small and decreasing size, and almost no episodes when writing at large and increasing amplitude also further corroborates the similarities between gait and upper limb freezing [8]. A novel finding of the current study is that these similarities are present even during a unimanual writing movement which differs from gait and bilateral finger tapping and drawing tasks used previously [12, 13]. The majority of the FOUL episodes were observed in patients who also experienced freezing of gait and significant, but moderate, correlations were found between the occurrence of FOUL and patients’ scores on the new freezing of gait questionnaire. As such, our results confirm previous work showing a correlation between freezing in the upper extremity and gait freezing and support the idea that both phenomena may share partially overlapping mechanisms [12]. Besides upper limb freezing, the patients with FOUL also presented with temporal and spatial abnormalities in the kinematic profile of their writing-like movements outside their freezing episodes. Unlike patients without FOUL, those with FOUL wrote significantly smaller and had a tendency to write faster even when no freezing was present. On the one hand, this may reflect the hastening phenomenon in the PD+FOG. On the other hand, it may be caused by increased anxiety or stress as patients were trying to prevent freezing episodes to occur. This behavior is in line with the movement abnormalities outside the freezing episodes that were reported during gait in patients with FOG [35–37]. Freezers were shown to have a decreased step length and greater loss of control of the frequency component of movement, predetermining freezing episodes to occur [35, 36]. Instructing patients to walk deliberately with small rapid steps was previously shown to be a sensitive way to provoke FOG in patients with gait freezing [7].

Our own and other studies [12, 15] also found FOUL episodes in a minority of PD patients without FOG. This either contradicts the above mentioned hypothesis of a common systemic deficit underlying different types of freezing or may be explained by the fact that freezing severity may gradually increase as the disease progresses, and that patients with only one type of freezing are prone to also develop freezing in the unaffected extremities later in the disease. This assumption, however, remains speculative and requires long term follow-up studies in patients with and without FOG. Patients in the PD-NoFOG and PD+FOG group were carefully matched for UPDRS-III scores, Hoehn and Yahr stages and MMSE scores, ruling out the possibility that the current findings were due to differences in disease severity or cognition. However, disease duration did differ and proved correlated to the FOUL occurrence.

Surprisingly, two FOUL episodes also occurred in one of the control subjects. On top of the freezing episodes, this person had a highly abnormal kinematic pattern, with more frequent stops than all other control subjects. Although this person reported no neurological or orthopedic problems, it cannot be ruled out that an undiagnosed deficit was at play. As well, it may be considered as an age-related behavioral deficit, which may be linked to reduced basal ganglia function [38, 39]. Alternatively, this finding may indicate that two voluntary stops were incorrectly identified as freezing episodes. This is, however, unlikely, as all voluntary stops were documented during data collection and interruptions of the kinematic trajectory that were not preceded by abnormalities in amplitude and frequency were always considered as stops instead of FOUL episodes.

In total, 50 freezing episodes were elicited in 32 out of 170 patient trials. The effectiveness and clinical relevance of the current writing task to elicit FOUL is even more striking given the fact that FOUL was elicited in the on-phase of the medication cycle. As such, this test reflects the difficulties patients may experience during daily living in contrast to studies conducted when off medication [12, 15]. Up to now, there is limited evidence showing that FOUL is, at least to some extent, dopa-responsive. Barbe et al. [40] showed a tendency that FOUL occurred more frequently during the off than during the on phase of the medication. The only study so far that investigated brain activity during FOUL reported hypoactivation in cortical motor and prefrontal areas during actual freezing, areas that likely become more activated upon dopaminergic medication intake [41]. Further research is needed on the neuroanatomical correlates of FOUL and its relation with FOG and levodopa intake. The current task, which was shown to be highly effective to provoke FOUL and is easy to use in a scanner environment, may be useful in this regard.

In addition, our repeated funnel task may also be a relevant clinical examination of patients with PD as it can easily be produced using paper and pencil. Alternatively, technology could be developed to offer this type of testing by means of a tablet or smartphone. Furthermore, current work is undertaken to develop a novel sensor system integrated with a digital pen to make the evaluation of handwriting in PD even easier in the future [42]. The repeated funnel task could be part of a general investigation of handwriting, which has been suggested to be a fast, reliable and cost-effective way to detect upper limb problems in patients with PD [43]. The duration of other tasks to investigate upper limb function, such as the UPDRS-items involving 10 repetitive hand movements, is often too short to elicit FOUL, which has been reported to rarely occur during the first 5 seconds of a movement [10]. As FOUL can be present during a wide range of repetitive ADL movements, such as writing, typing, tooth brushing and playing an instrument, we believe that an adequate diagnosis and therapeutic approach of this symptom is warranted.

In summary, we have used a novel writing paradigm with potential ecological and clinical value that effectively provokes upper limb freezing in approximately 50% of patients with FOG while on medication. Corroborating findings of gait research, the current study supports a core problem in amplitude control underlying FOUL, both in maintaining as well as flexibly adapting the cycle size which is possibly aggravated by a deficit in set shifting.

## Supporting Information

S1 Dataset Patient characteristics and outcome measures. (XLSX)
